# Contribution of SPECT/CT for sentinel node localization in patients with ipsilateral breast cancer relapse

**DOI:** 10.1007/s00259-016-3545-8

**Published:** 2016-10-27

**Authors:** Pablo Borrelli, Maarten L. Donswijk, Marcel P. Stokkel, Suzana C. Teixeira, Harm van Tinteren, Emiel J. Th. Rutgers, Renato A. Valdés Olmos

**Affiliations:** 1 Department of Nuclear Medicine, The Netherlands Cancer Institute, Antoni Van Leeuwenhoek Hospital, Plesmanlaan 121, 1066 CX Amsterdam, The Netherlands; 2 Nuclear Medicine, Medical Imaging Clinical Area, Hospital La Fe, Valencia, Spain; 3 Department of Biometrics, The Netherlands Cancer Institute, Antoni Van Leeuwenhoek Hospital, Amsterdam, The Netherlands; 4 Department of Surgery, The Netherlands Cancer Institute, Antoni Van Leeuwenhoek Hospital, Amsterdam, The Netherlands; 5 Nuclear Medicine Section and Interventional Molecular Imaging Laboratory, Department of Radiology, Leiden University Medical Centre, Leiden, The Netherlands

**Keywords:** SPECT/CT, Sentinel node, Lymphoscintigraphy, Recurrent breast cancer, Radioguided surgery

## Abstract

**Background:**

In recent years repeat sentinel node (SN) biopsy has been proven to be feasible in local breast cancer recurrence (LBCR). However, in these patients SNs outside the ipsilateral axilla are frequently observed. This study evaluates the contribution of SPECT/CT for SN localization and surgical adjustment in LBCR patients.

**Methods:**

SN biopsy was performed in 122 LBCR patients (median age 60.5 years, range 24–87), enrolled from August 2006 to July 2015. Median disease-free time lapse was 109.5 months (range 9–365). Axillary lymph node dissection (ALND) had previously been performed in 55 patients, SN biopsy in 44, both techniques in 13 and fine-needle aspiration in 10. Primary breast cancer treatment included radiotherapy in 104 patients (85.3 %) and chemotherapy in 40 (32.8 %). Preoperative lymphatic mapping, using planar scintigraphy (PS) and SPECT/CT included report of SN location according to lymph node territory. In case of a territorial PS-SPECT/CT mismatch, surgery was adjusted according to SPECT/CT findings.

**Results:**

SPECT/CT SN visualization rate was higher than PS (53.3 % vs. 43.4 %, p n.s.) with, in total, 19 additional SN (118 vs. 99, p n.s.). PS-SPECT/CT territory mismatch, found in 60 % (39/65) of patients with SN visualization, led to surgical adjustment in 21.3 % (26/122) of patients. The SN procedure was finally performed in 104 patients resulting in a 65.7 % surgical retrieval rate with a total of 132 removed SNs (1.86/patient). SN metastases were found in 17/71 patients (23.9 %), in 16 of them (94 %) in ipsilateral basins outside the axilla or in the contralateral axilla.

**Conclusion:**

Using SPECT/CT there is a trend to visualize more SNs in LBCR, providing at the same time important anatomical information to adjust intraoperative SN procedures. The addition of SPECT/CT to the standard imaging protocol may lead to better staging mainly in patients presenting drainage outside the ipsilateral axilla.

## Background

Breast cancer is the most frequent cancer in women with 1.67 million new cases in 2012, ranking second in frequency worldwide [1]. The highest incidence is in Western Europe, reaching over 96 new cases per 100,000. Breast cancer mortality dropped over the last few decades due to earlier diagnoses by screening, increasing patients awareness and improved comprehensive care [2]. To achieve individualized patient treatment one should take into account staging, biological cancer information, personal preferences (patient and treating physician) among other variables. Nowadays, a broad therapeutic arsenal is at our disposal: different forms of surgery, radio- and chemotherapy, endocrine therapy, and more specific targeted therapy [3].

Lymph node status is the most important prognostic factor in primary breast cancer [4]. This is acknowledged in many guidelines, as in the most recent ESMO guidelines in 2015 [5]. It is likely that nodal status is also prognostic in locally recurrent breast cancer. Therefore, knowledge of nodal status influences regional treatment strategies, particularly radiotherapy, and may also enforce the indication for adjuvant systemic (re-)treatments with curative intention [6]. Since the introduction of the SN procedure in breast cancer planar scintigraphy (PS) has been the basis for lymphatic mapping and has proven to be an accurate tool for SN localization in primary cancer, leading to detection rates of over 97 % and 98 % in series of 4800 [7] and 3681 patients [8], respectively.

Although relevant medical progress was reached in diagnosing and treating breast cancer in the last decade, locoregional recurrence is still an issue after a full set of treatment, ranging from approximately 3 % to near 30 % when associated with distant metastasis [9, 10]. Contrary to high 5-year survival rates known for primary breast cancer, relapse breast cancer has a high mortality rate. Nonetheless, life expectancy is improving due to follow-up care after treatment, and relapses are usually detected in asymptomatic stages [11]. Ten-year survival of breast cancer patients after local recurrence was around 20 % in the 1980s and 1990s [12] and increased to approximately 35-45 % in more recent years [13]. The last Dutch revision in 2015 on survival rates after locoregional relapse showed a promising 49 % survival rate at 10 years [11].”

Against this background, the sentinel node (SN) procedure has been introduced in breast cancer patients experiencing a local relapse. This appears to be technically feasible, although detection rates vary significantly from 62.1 % to 80.4 % [4–6, 14, 15] among several studies. Although several factors such as tumour size and patient age have been described to alter lymphatic drainage in patients at primary breast cancer presentation [16] in LCBR patients, previous treatment of the primary cancer has incorporated additional variables that are even more anatomically and physiologically destructive, including surgery of both the axillary region and primary tumour, systemic treatments and radiation therapy. This previous treatment frequently leads to a more complex and multidirectional lymphatic drainage with a high incidence of SNs outside the axilla suggesting the need for accurate localization imaging modalities like single photon emission computed tomography in conjunction with computed tomography (SPECT/CT) [17].

Early in 2015 the results from a multicentre prospective study on the additional value of SPECT/CT over PS in various malignancies, including primary breast cancer, coordinated by the International Atomic Energy Agency (IAEA) were published. On the basis of 1.182 enrolled breast cancer patients, SN visualization increased from 87.6 % for PS to 91.3 % when SPECT/CT was used additionally, and in 44 patients only visualization on SPECT/CT enabled the SN procedure [18]. SPECT/CT led to adjustments in anatomical SN localization and surgical management in 17 % of the patients. So far no studies have addressed the SPECT/CT impact in a similar manner for breast cancer relapses.

The aim of this study was to evaluate whether the use of SPECT/CT improved both visualization, and anatomical localization of the SN, and whether this additional information may have had impact on the surgical approach and consequent retrieval of SN in patients with local breast cancer relapse (LBCR) scheduled for SN biopsy.

## Material and methods

At the Netherlands Cancer Institute – Antoni van Leeuwenhoek Hospital LBCR patients were incorporated to the SN procedure from 2006 onward using both PS and SPECT/CT, together with the introduction of SPECT/CT in the protocol for lymphatic mapping in untreated breast cancer patients [19]. All patients were restaged to exclude metastases at distance following the Dutch breast cancer guidelines, which include total body scanning with ^18^F-FDG PET/CT. After giving informed consent from August 2006 to October 2015, 122 consecutive patients with repeat SNB for local breast cancer relapse were studied following the protocol including SPECT/CT. Their data were analysed retrospectively.

All patients were female with a median age of 60.5 years (range 24 – 87) at time of LCBR and a median disease-free time interval of 109.5 months (range 9 – 365). Primary tumour surgery at initial presentation was breast sparing in 117 patients (96 %) and mastectomy in 5 (4 %). Lymphatic status at first surgery was assessed with SNB procedure in 44 patients. ALND alone was performed in 55 patients, 13 patients had ALND after a positive SN. In 10 patients the N-stage was assessed using clinical examination and ultrasound guided fine needle aspiration. Metastases were found in 32 out of 112 patients who had undergone lymphatic surgery. Systemic treatment was applied in 40 (32.8 %) patients and radiotherapy either to the breast, axilla or both regions in 104 (85.3 %). Hormonal treatment information was not available for all patients and could therefore not be included in this evaluation. Patient characteristics are shown in Table 1.

**Table 1:** Description of patient characteristics, primary tumor treatment, characteristics of relapse tumour, and activity and location of radiotracer injection

Characteristic		Median	Range
Number of patients	122		
Sex			
Female	122		
Male	0		
Age at relapse (years)		60.5	24 - 87
Months to relapse		109.5	9 - 365
Injected dose (MBq)		126.13	63.94 - 250.63
Injection location			
Intratumoral	113		
Peritumoral	3		
Periareolar	2		
Subcutaneous	4		
Relapse tumor size		15 mm	3 - 70 mm
Primary treatment			
• Surgery			
Breast conserving surgery	117	95.9 %	
Mastectomy	5	4.1 %	
ALND	68	55.7 %	
SNB	57	46.7 %	
ALND + SNB	32	26.2 %	
• Radiotherapy	104	85.2 %	
• Chemotherapy	40	32.8 %	

**Fig. 1:**
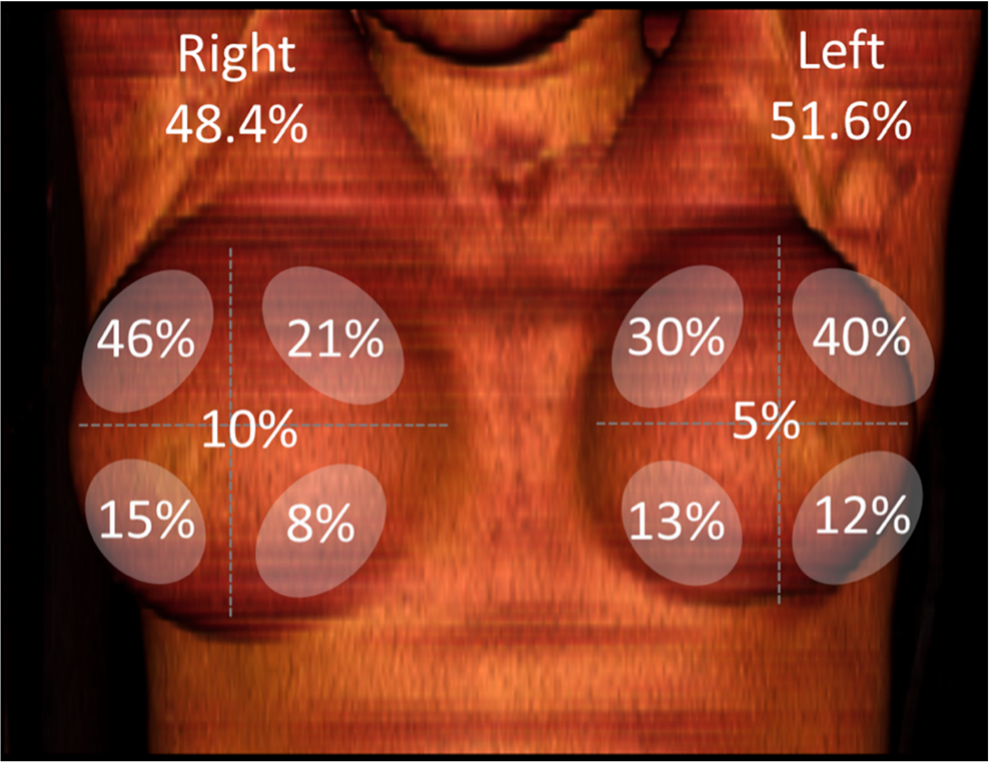
Distribution of tumour relapse according to imaging technics and surgical findings. Four quadrants were considered and the figures depicted in the center of each breast stand for retroareolar tumours

Histological evaluation of the LBCR revealed ductal carcinoma in 99 patients (81.2 %) and lobular in 18 (14.8 %). In another five cases (4 %) the tumour type was adenocarcinoma (not further specified), intracystic carcinoma, mucinous, metaplastic and papillary. In 59 patients tumours were located in the left breast and in 63 in the right. The locations of the recurrences are shown in Fig. 1. Tumour sizes ranged from 3 to 70 mm with a median size of 15 mm. Hormone positive receptors were present in 80 tumours, while 22 were triple negative. Her-2 was positive in 12 patients.

## Sentinel node procedure

In 113 patients (92.6 %) ^99m^Tc-Nanocolloid (Nanocoll^©^; GE Healthcare, Eindhoven, Netherlands) was administered by intratumoural injection, the standard procedure in our hospital; either palpation-based or image-guided (ultrasound, stereotactic, or using ^125^I-seed guided freehand-SPECT tumour localization). Image-guided intratumoural injection was performed in 43 patients (35 %). In nine patients (7 %) the injection was made either subcutaneously (n = 4), periareolar (n = 3), or peritumourally (n = 2). Median injection activity was 126.1 mBq (range 63.9-250.6). This dose matches the recommendation for a 2-day SN procedure using intratumoral tracer administration as validated in our institution [20].

Early planar scintigraphy was made at an average of 23 minutes after injection, and a late planar scintigraphy (PS) at an average of 4 h. This is in agreement with the published EANM guidelines for SN procedures in breast cancer [21]. PS comprised 5-min static images in anterior and lateral/oblique projections, 256 × 256 matrix, and zoom factor 1.0. A ^57^Cobalt flood source was used to delineate the body contour. SPECT/CT was made at an average of 4.42 h after injection using a dual-head SPECT/CT gamma camera (Symbia T®, Siemens, Erlangen, Germany). SPECT parameters were: 128 × 128 matrix, zoom factor 1.0, and 180 degrees rotation with 20 views per head (30 s per view). CT (130 Kv, 40 mAs, B30s kernel, 2 mm axial reconstruction) was used for attenuation correction and anatomical localization. For image reading SPECT, CT, and fused SPECT/CT were displayed using orthogonal multiplanar reconstruction, maximum intensity projection and volume rendering.

As SNs were considered all lymph nodes with afferent lymphatic vessel draining directly the injection site, or, in cases of multiple nodes appearing with no afferent lymphatic vessels on the lymphoscintigram, the first node appearing in each basin [22]. The number of SNs and their draining basin localization were evaluated separately on PS and SPECT/CT images to establish the incremental value of SPECT/CT over PS with respect to additional SNs or territorial mismatch following the criteria applied in a multicenter International Atomic Energy Agency trial [18]. The anatomical territories considered for the evaluation were the following: axillar level I, II, and III, intramammary, interpectoral, clavicular, internal mammary, all of them either ipsilateral or contralateral. Partial mismatch was considered when a patient showed a sentinel node that was correctly depicted and found, but also had one or more sentinel nodes that were mismatches. Complete mismatch between PS and SPECT/CT was considered whenever sentinel nodes and/or anatomical location in a patient were totally discordant with each other.

Surgery was performed either the same day or the day following the radiotracer injection. Intra-operative SN detection was performed with a conventional gamma probe (Neoprobe®, Neoprobe Corporation, Dublin, OH, USA). In addition, patent blue dye was injected after anaesthesia induction in 76 (62.3 %) patients depending on the leading surgeon preoperative decision. In principal, all SNs visualized on PS and SPEC/CT were to be removed. Adaptation of the surgical procedure for SN biopsy was made in all cases with territorial mismatch unless this concerned only a difference in axillary levels (levels 1 to level 2, for example).

Factors associated with the SN detection were analysed using logistic regression and statistical significance was established at p < 0.05. The following variables related to prior primary tumour were included in the analysis: age, previous ALND or SNB, radiotherapy, chemotherapy, and relapse variables as time to relapse, tumour size, total activity administered, image guided injection or not. All of these variables were added in order to identify possible causes and confusion factors of altered sentinel node detection rates. Continuous variables were checked for linearity and categorized if shown to be non-linear. The analysis was performed using R version 3.2.3 (2015-12-10).

## Results

SPECT/CT visualized SNs in 65 patients (53.3 %) and PS in 53 patients (43.4 %). This resulted in a 9.8 % visualization rate improvement (p n.s.). In the present study early planar imaging visualized SNs in 22 patients (18 %), whereas on delayed PS SNs were visible in 53 patients (43.4 %), an increment of 25.4 %. A total of 118 SNs were visualized on SPECT/CT and 99 on PS. Overall, SN visualization rate was 63.2 % (43/68) for patients with previous ALND with or without SN biopsy, and 38.6 % (17/44) for patients with previous SN biopsy alone. Results from a univariable (crude OR) and multivariable (adjusted OR) logistic regression model are described in Table 2. Older age showed to be the most important factor in the multivariable model and was linearly associated with a decreased detection rate (OR 0.95, 95%CI: 0.90-0.99, unit is years). Alternative lymphatic drainage (SN visualized outside the ipsilateral axilla) was found in 43 % (28/65) of the patients and contralateral SN visualization was present in 26 % (17/65) of the cases. Early PS visualized 34 SNs in 22 patients (18 %).

**Table 2:** Univariable logistic regression models with testing for nonlinear effects

Variable	Median (Q1-Q3)/Freq	OR	p	global p	Nonlinear
Time to relapse	110 (62;228)			0.032	yes
Age	60 (51;67)	0.950		0.004	no
Tumor Size	15 (11;20)	1.000		0.881	no
ALND				0.013	
No	54	1.000			
Yes	68	2.502	0.014		
SNB				0.112	
No	65	1.000			
Yes	57	0.559	0.113		
Radiotherapy				0.020	
No	18	1.000			
Yes	104	0.275	0.031		
Chemotherapy				0.513	
No	82	1.000			
Yes	40	1.289	0.514		
Total activity	126 (116:135)	0.980		0.106	no
Image guided				0.427	
No	79	1.000			
Yes	43	1.354	0.428		

**Fig. 2:**
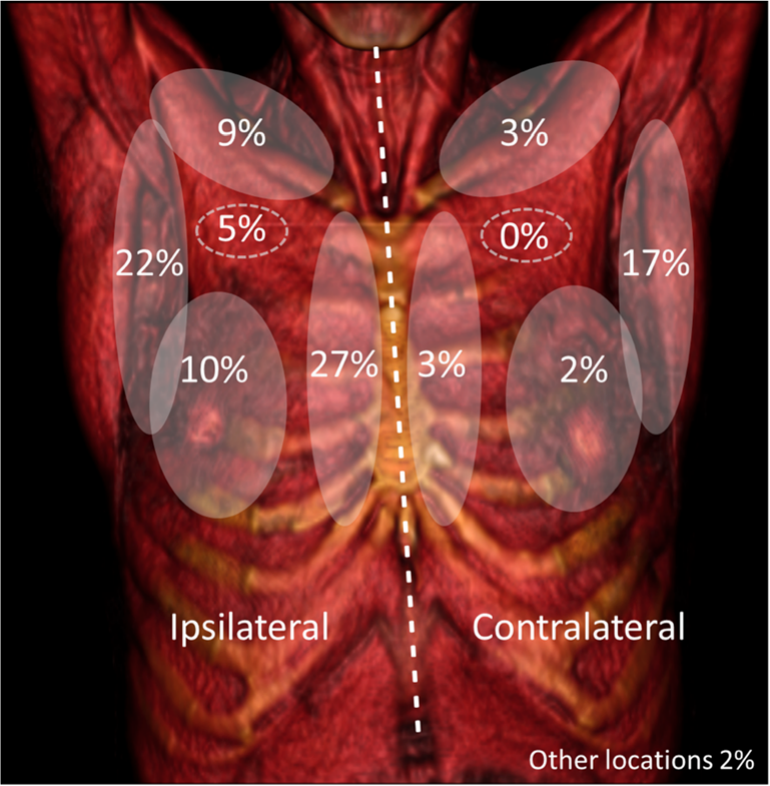
SN localization distribution using SPECT/CT imaging. Areas depicted in clear grey are: axillar, clavicular, internal mammary chain, and intramammary; bilateral areas with discontinued lines are depicting intrapectoral nodes

SN distribution based on imaging is summarized in Fig. 2.

**Fig. 3:**
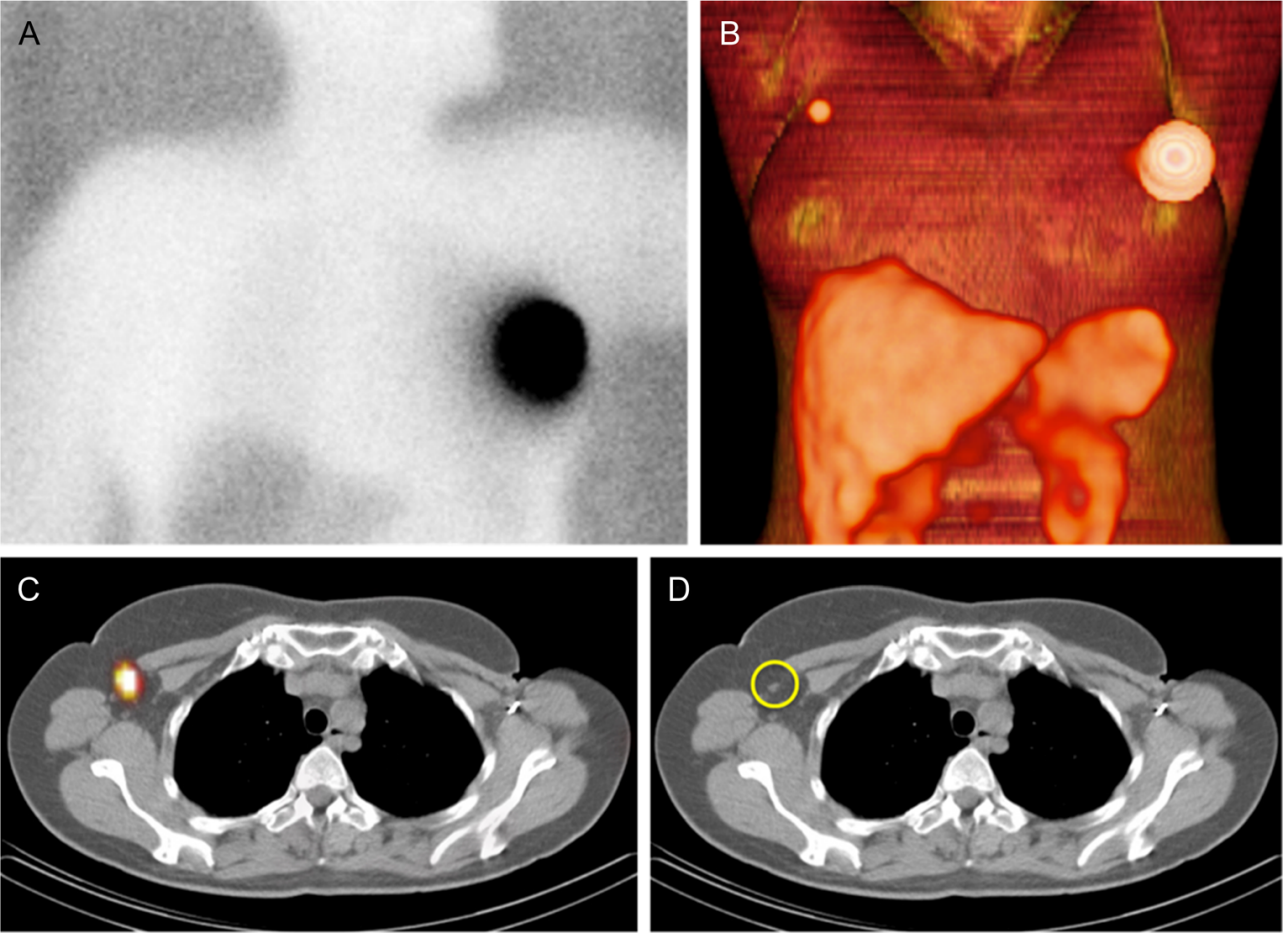
A 48-year-old woman with a ductal carcinoma relapse in the left breast, without SN visualization on planar image (**a**). Volume rendering of SPECT/CT (**b**) shows a focus in level 1 of the contralateral axilla (figure **c**) corresponding with a non-enlarged lymphatic node (circle) on CT (**d**)

**Fig. 4:**
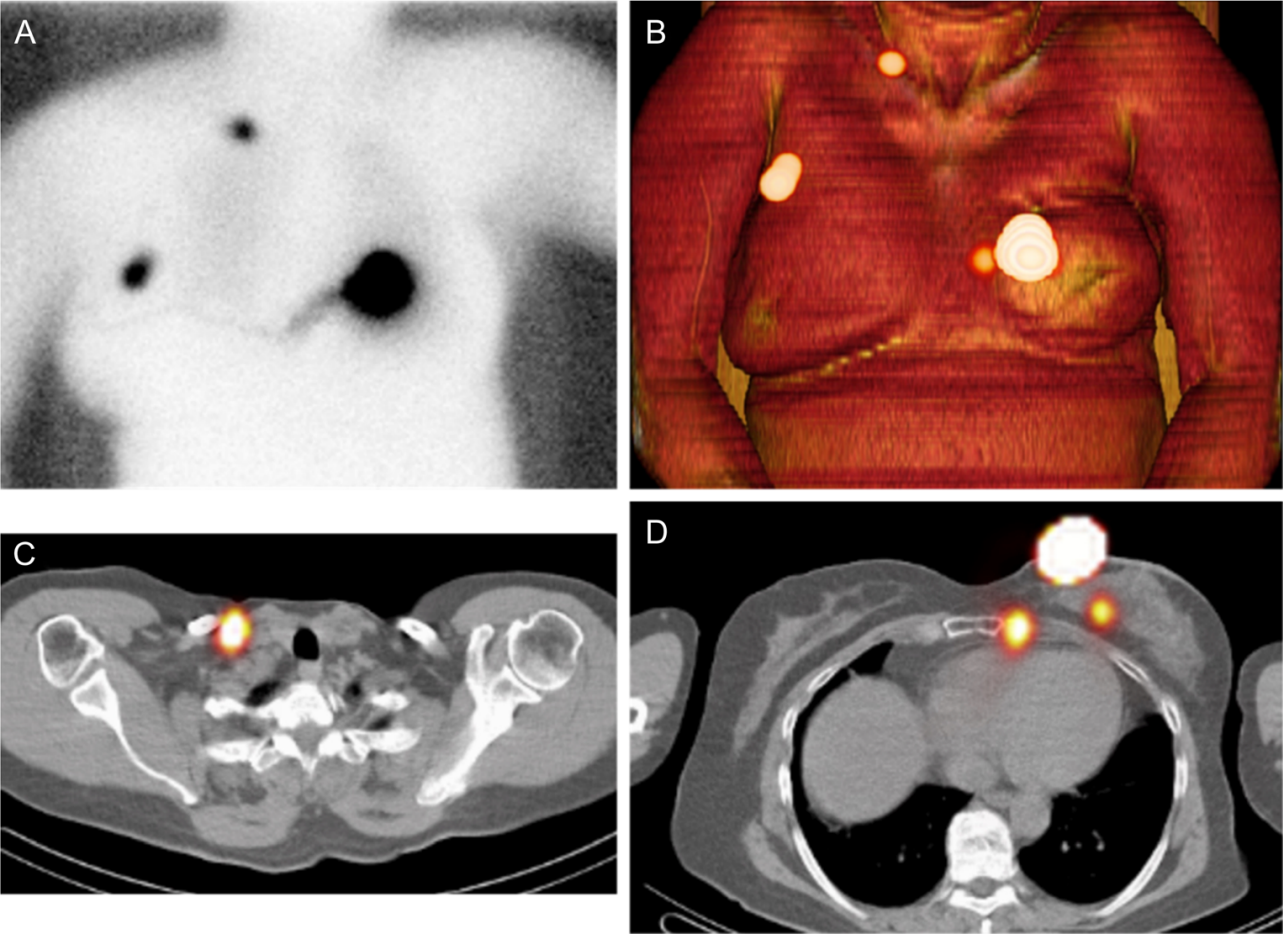
A 61-year-old woman with ductal carcinoma relapse in the left breast. (**a**) Late planar scintigraphy shows a lymphatic duct towards the contralateral axilla. Also, a supraclavicular SN is visualized. (**b**) Volume rendering shows both axillary and supraclavicular SNs together with a focus medial to the injection site and altered anatomical configuration of the left breast area due to primary treatment. (**c**) Supraclavicular SN on the right. (**d**) Dorsal of the injection site two SN are visualized on SPECT/CT, one intramammary and one intercostal, not visualized on planar scintigraphy. Histopatology demonstrated micrometastasis in the axillary node whereas the intramammary, intercostal and supraclavicular SN were negative

**Fig. 5:**
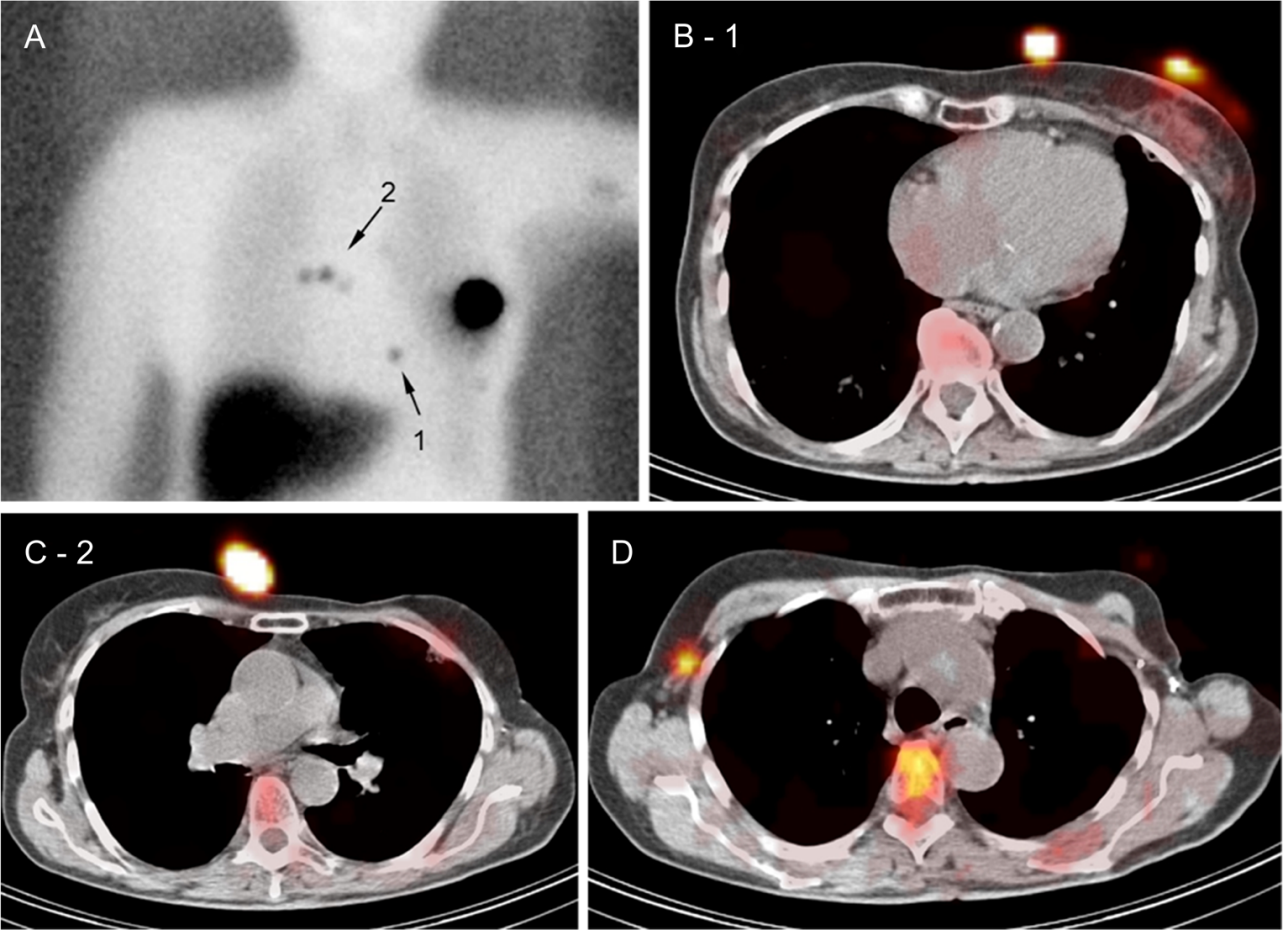
A 77-year-old woman with ductal carcinoma relapse in the left breast. Planar scintigraphy shows bilateral foci parasternally (**a**), arrows number 1 and 2. SPECT/CT (**b**) shows the first lower left focus as skin contamination. Also, the focus parasternally right appeared to be due to skin contamination (**c**). Note that a SN which was not visualized on planar scintigraphy is seen in level 1 of the right axilla (contralateral) on SPECT/CT (**d**). This axillary SN was negative for metastasis

Territorial mismatches between PS and SPECT/CT were observed in 39 out of the total of 65 patients (60 %) with SN visualization. The most common identified territorial mismatches were: 1) no visualization on PS accompanied by SPECT/CT visualization in the ipsilateral axilla; this was found in seven cases (17.9 %). 2) Drainage to contralateral axilla not visualized on PS, but well visualized on SPECT/CT in five patients (12.8 %) (Fig. 3). 3) Drainage to ipsilateral intramammary chain in five patients (12.8 %) not visualized on PS and well visualized on SPECT/CT (Fig. 4). 4) In three patients (7.7 %) putative SN visualization on PS appeared to be due to tracer contaminations as shown on SPECT/CT (Fig. 5)

Surgical SN approach was adjusted using final SPECT/CT information in 26 out of 122 patients (21.3 %). The SN procedure was not performed in 18 patients with previous ALND and non-visualization at preoperative imaging. The intraoperative SN procedure was performed in 104 patients but only in 71 of them the SN was identified and removed. This resulted in a 68.3 % surgical SN resection rate. In seven of these patients the SN could be identified using blue dye only. Details concerning detection by gamma probe and/or blue dye are given in Table 3.

**Table 3:** Surgical sentinel node procedures and detection findings

N (patients)	
Total scheduled SN procedures	122
Total performed SN	104
Detected	71 (68.3 %)
Radioactive	64
Blue	29
Only radioactive	42
Only blue	7

At histopathology, SN metastases were found in 17/71 patients (23.9 %): eight with macrometastasis and nine with micrometastasis. SN metastases were located outside the ipsilateral axilla in 64.7 % of the cases (11/17) and in the contralateral axilla in 29.4 % (5/17).

ALND was performed in 20 patients; with a total of 223 LN retrieved, median 11.2 nodes per patient (range 2 – 27). In 15 patients (75 %) histopathology showed no further LN involvement, in 5 (25 %) macrometastases were found. ALND did not reveal metastases in any of the patients with a negative SN.

After surgical treatment of the relapse 60 patients received hormone therapy, 43 chemotherapy and 30 radiotherapy, 38 patients received no further treatment.

## Discussion

The results of the present study show that additional SPECT/CT may play an essential role in patients with breast cancer recurrence scheduled for the SN procedure.

From a clinical point-of-view probably the most important contribution of SPECT/CT is the information provided to adjust the surgical approach. This concerned over one in every five breast cancer relapse patients in this study (21,3 %). The results achieved in our series seem to be in a good agreement with data from the last IAEA trial (17 % vs. 21 %); however, this small difference could be justified, firstly, due to the difference in population, and secondly, due to the complicating factors associated to local recurrent cancer mentioned in the introduction [18]. The adjustment of the surgical approach may also lead to a better restaging in LCBR patients as in the present study SN metastases were located in ipsilateral territorial basins outside the axilla or in the contralateral axilla in 16 out of 17 (94 %) patients.

This surgical approach adjustment was particularly crucial in three patients in whom SPECT/CT correctly identified putative SN visualization due to contamination artefacts and avoided SN surgery. Similar artefact-related cases have been reported in the multicentre IAEA SN trial, where 41 (out 1182) unnecessary SN surgeries could be avoided [18].

The superior anatomical information provided by SPECT/CT was crucial to establish a 60 % territorial mismatch evidenced after the incorporation of SPECT/CT to the conventional protocol based on PS. The anatomical information provided by SPECT/CT enabled nuclear physicians to accurately localize SN in relation to specific lymph node basins and anatomical landmarks.

For the present study static delayed images were acquired 3–4 h after intratumoral tracer injection. This delayed point of time is based on the radiotracer biodistribution, which is completed at that moment (see the above mentioned reference). Delayed imaging (planar and SPECT/CT) is important to exclude eventual drainage outside of the ipsilateral axilla. Theoretically, in patients with local relapse tracer migration from the injection site to SN is slower than in patients without previous surgery and/or radiotherapy of the breast/axilla. This was also observed in our series, in agreement with a previous study concerning SN biopsy in patients with treated breasts [17].

Finally, SPECT/CT in this series led to detect more SNs with almost 10 % improvement in the detection rate when compared with conventional PS. These results are higher than those reported in an earlier study in our hospital by van der Ploeg et al. [23] and the more recently published multicenter IAEA SN trial, both considering only untreated primary breast cancer patients [18]. However, this detection improvement related to SPECT/CT did not reach statistical significance and probably a larger series of patients will be needed for a definitive validation of the role of SPECT/CT.

Many factors affecting SN visualization have been largely discussed in other papers. Some examples are: body mass index (BMI), age at time of relapse, previous ALND, and radiotherapy [16]. Also, recently, Vugts et al. analyzed multiple variables and found that only previous radiotherapy had a significant association with lower SN detection rates in LBCR patients [24]; this could also explain the lower detection rates in our series in comparison with the aforementioned series [14, 15, 25–27].

An interesting concept and something probably worth of further study is that there seems to be a tendency of lymphatic regeneration after ALND and/or radiotherapy. A non-linear association between time to relapse and SN detection rates supports this idea. Cordoba et al. also suggested such a concept [25]. The results in our series also show a nonlinear association between the period of “recovery” (relapse-free period) and an improvement in detection rate (Table 2). Patients with breast cancer relapse within 10 years had a 46 % detection rate versus 62 % for patients with a relapse-free period of more than 10 years. Blum et al. also proved that neo-lymphatic vascularization was present in mice which had undergone ALND-like surgery [28].

## Conclusion

In conclusion, although not reaching statistical significance the addition of SPECT/CT to the standard imaging protocol for lymphatic mapping and SN localization in breast cancer recurrence patients appears to improve detection of sentinel nodes and their anatomical localization. This all may contribute to the adjustment of the surgical sentinel node approach and a better staging mainly in patients presenting drainage outside of the ipsilateral axilla.
